# The Role of Circadian Rhythm in Neurological Diseases: A Translational Perspective

**DOI:** 10.14336/AD.2023.0921

**Published:** 2024-08-01

**Authors:** Wanbin Huang, Jiabin Zong, Yu Zhang, Yanjie Zhou, Lily Zhang, Yajuan Wang, Zhengming Shan, Qingfang Xie, Ming Li, Songqing Pan, Zheman Xiao

**Affiliations:** ^1^Department of Neurology, Renmin Hospital of Wuhan University, Wuhan, Hubei, China; ^2^Department of Neurology, Union Hospital, Tongji Medical College, Huazhong University of Science and Technology, Wuhan, China.

**Keywords:** circadian rhythm, biological clock, neurological diseases, translational medicine

## Abstract

Intrinsic biological clocks drive the circadian rhythm, which coordinates the physiological and pathophysiological processes in the body. Recently, a bidirectional relationship between circadian rhythms and several neurological diseases has been reported. Neurological diseases can lead to the disruption of circadian homeostasis, thereby increasing disease severity. Therefore, optimizing the current treatments through circadian-based approaches, including adjusted dosing, changing lifestyle, and targeted interventions, offer a promising opportunity for better clinical outcomes and precision medicine. In this review, we provide detailed implications of the circadian rhythm in neurological diseases through bench-to-bedside approaches. Furthermore, based on the unsatisfactory clinical outcomes, we critically discuss the potential of circadian-based interventions, which may encourage more studies in this discipline, with the hope of improving treatment efficacy in neurological diseases.

## 1. Introduction

Therapies and treatment strategies remain limited for a considerable proportion of disabling neurological diseases owing to their complicated and unclear pathophysiology [1]. Poor clinical outcomes further necessitate the development of more effective treatments. An in-depth understanding is thus needed to offer novel perspectives on neurological disease pathologies.

In 1929, an observational study described the dependence of seizures on diurnal rhythms [2]. Over a period of 6 months, a total of 2524 seizures were documented in 66 individuals by the researchers. It was discovered that patients with epilepsy (PWE) had an increased likelihood of experiencing seizures at specific times during the day, which subverted the previous belief that seizures are random. Over time, the circadian patterns in the clinical presentations of neurological diseases were widely reported [3, 4]. Although these phenomena have been extensively described over decades, the underlying correlation needs to be clarified.

This review initially presents a summary of the existing clinical and experimental proof concerning the impact of circadian regulation on neurological disorders. Next, we evaluated the potential mechanism of how the circadian rhythm regulates the pathophysiology of neurological diseases and vice versa exhibiting a bidirectional relationship. Finally, we have discussed the therapy and management strategies based on data available from circadian biology to help toward developing new therapeutic targets and identifying predictive biomarkers. Circadian-based interventions could help design personalized and precision medicine for patients. Chronotherapy has achieved initial success in the treatment of neurological diseases, suggesting that circadian biology can provide successful treatment strategies.

## 2. Overview of the circadian rhythm

**Figure 1.**
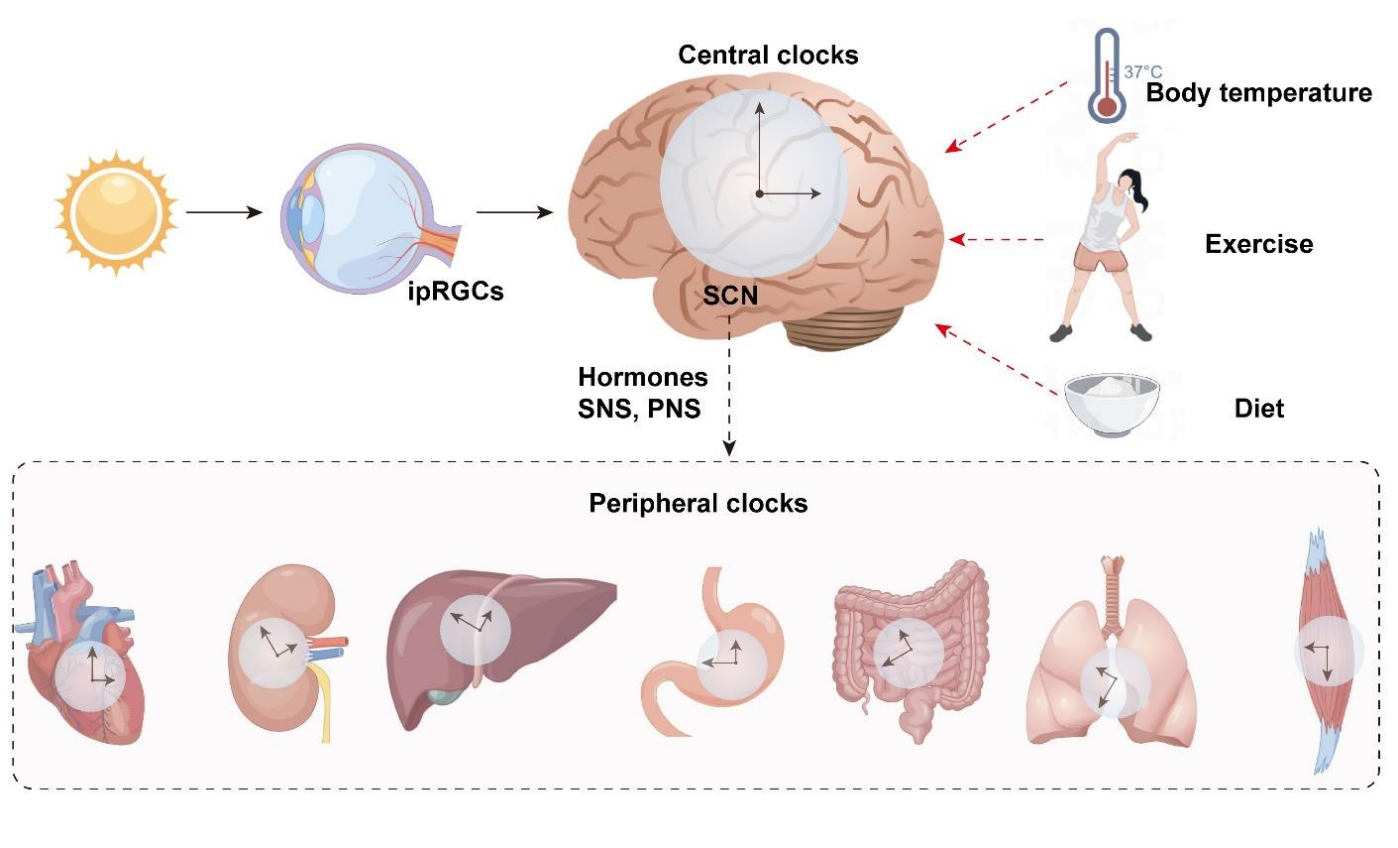
**Schematic of the human circadian system**. The circadian rhythm comprises the central clock located in the SCN. The intrinsic circadian system is regulated by zeitgebers, including light, body temperature, exercise, and diet. The central clock is synchronized with the external environment through the zeitgebers. In addition, all organs and tissues have their own clocks. The SCN regulates the peripheral clocks and synchronizes them with the central clock through hormones, the sympathetic nervous system (SNS), and the parasympathetic nervous system (PNS).

Physiologically, the mammalian biological clock, which controls the circadian rhythm cycle, consists of the central master clock and peripheral clock. The central master clock is located in the suprachiasmatic nucleus (SCN) of the hypothalamus. The SCN receives external light signals from the intrinsically photosensitive retinal ganglion cells (ipRGCs) through the retinohypothalamic tract and simultaneously communicates with other brain regions [5]. The SCN acts as a pacemaker that drives the circadian rhythms, such as sleep-wake, hormones, and body temperature [6]. The peripheral clock is widely distributed in various organs and tissues, and is regulated by the SCN through body fluids and the nervous system (e.g., cortisol rhythm and melatonin rhythm) [7]. The circadian rhythm is not only regulated by the intrinsic clock but also affected by external stimulation factors, such as light, diet, exercise, and temperature [7]. The internal circadian rhythm and external stimulation factors work together to synchronize the clock with the external environment in an organism (Fig. 1). Under the influence of the central clock, the circadian rhythm of each tissue and organ is fine-tuned by the peripheral clock and external signals.

**Figure 2.**
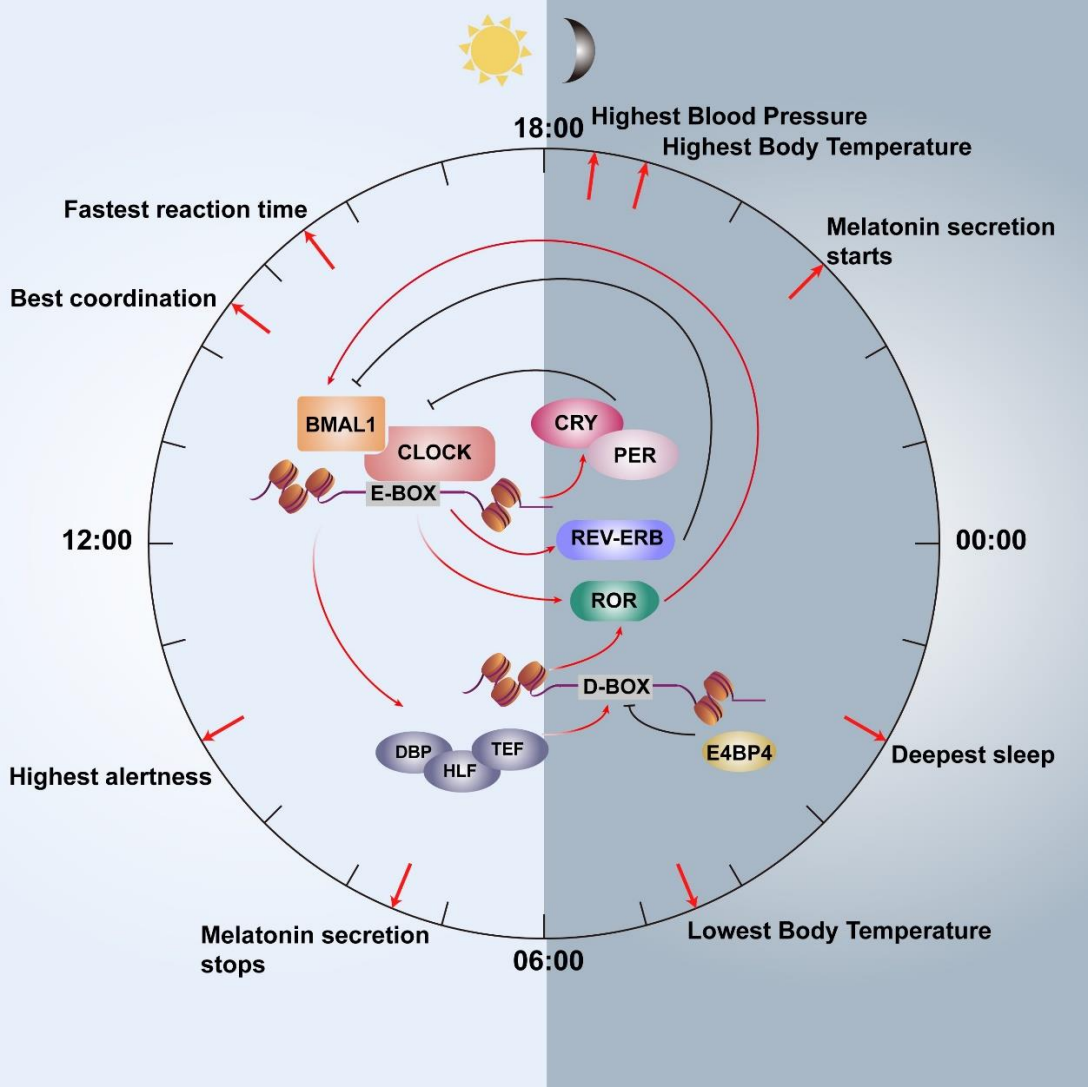
**Core molecular mechanisms underlying the circadian oscillation**. The core circadian molecular mechanisms involve a transcriptional feedback loop formed by several CCGs. The CLOCK-BMAL1 dimer binds to the E-box and promotes the transcription of CCGs. The PER-CRY heterodimers repress the CLOCK-BMAL1-mediated transcription. Meanwhile, the expression of BMAL1 is also governed by REV-ERB and RORs. The transcription of D-box-containing genes, such as RORs, is regulated by PAR bZIP transcription factors (DBP, HLF, and TEF) and E4P4.

At the molecular level, the circadian rhythm is regulated by a group of clock genes, which control the expression of output genes throughout the body. The clock genes form an interconnected transcriptional-translational feedback loop. The core of this feedback loop includes the transcription factors brain and muscle aryl hydrocarbon receptor nuclear translocator-like protein-1 (BMAL1) and circadian locomotor output cycles kaput (CLOCK) that heterodimerize, bind to the cis transcription element E-box on DNA, and promote the transcription of clock-controlled genes (CCGs) [8]. This marks the start of the feedback loop. PER and CRY proteins made by period genes (PER1, PER2, and PER3) and cryptochrome genes (CRY1 and CRY2), respectively, are also part of the CCGs feedback loop. These 2 proteins are produced in the cytoplasm and form heterodimers, translocate into the nucleus, and accumulate. The heterodimeric complex interacts with BMAL1/CLOCK to inhibit the transcription process [9]. In addition, retinoic acid receptor-related orphan receptors (RORs) and nuclear receptors REV-ERB are involved in the transcriptional regulation of the feedback loop. They can act on the ROR-response element (RRE) in the BMAL1 promoter, thereby mediating the expression oscillation of BMAL1 [10]. In addition, the proline and acidic amino acid-rich basic leucine zipper (PAR bZIP) transcription factors and the E4 promoter-binding protein 4 (E4BP4) are output mediators in the transcriptional regulation of the clock gene. The PAR bZIP transcription factors encompass albumin D-site-binding protein (DBP), hepatic leukemia factor (HLF), and thyrotrophic embryonic factor (TEF). They can bind to the cis transcription element D-box to regulate the transcription of CCGs, such as RORs [11, 12]. Together, the mechanisms allow the expression of clock proteins in a 24-hour cycle, thus mediating the circadian rhythm in mammals (Fig. 2).

## 3. Epilepsy

PWE have abnormal brain activity between seizures, known as interictal epileptiform activity (IEA), in addition to abnormality in an electroencephalogram (EEG) during seizures [13]. Emerging evidence has shown multiple timescales in seizures and IEA cycles including circadian (about 24 hours), multidien (about weeks to months), and circannual (about 1 year) [14]. Our main focus is the correlation between the circadian cycle and epilepsy.

### 3.1 Circadian rhythm of epilepsy

#### 3.1.1 Circadian patterns of epilepsy

**Figure 3.**
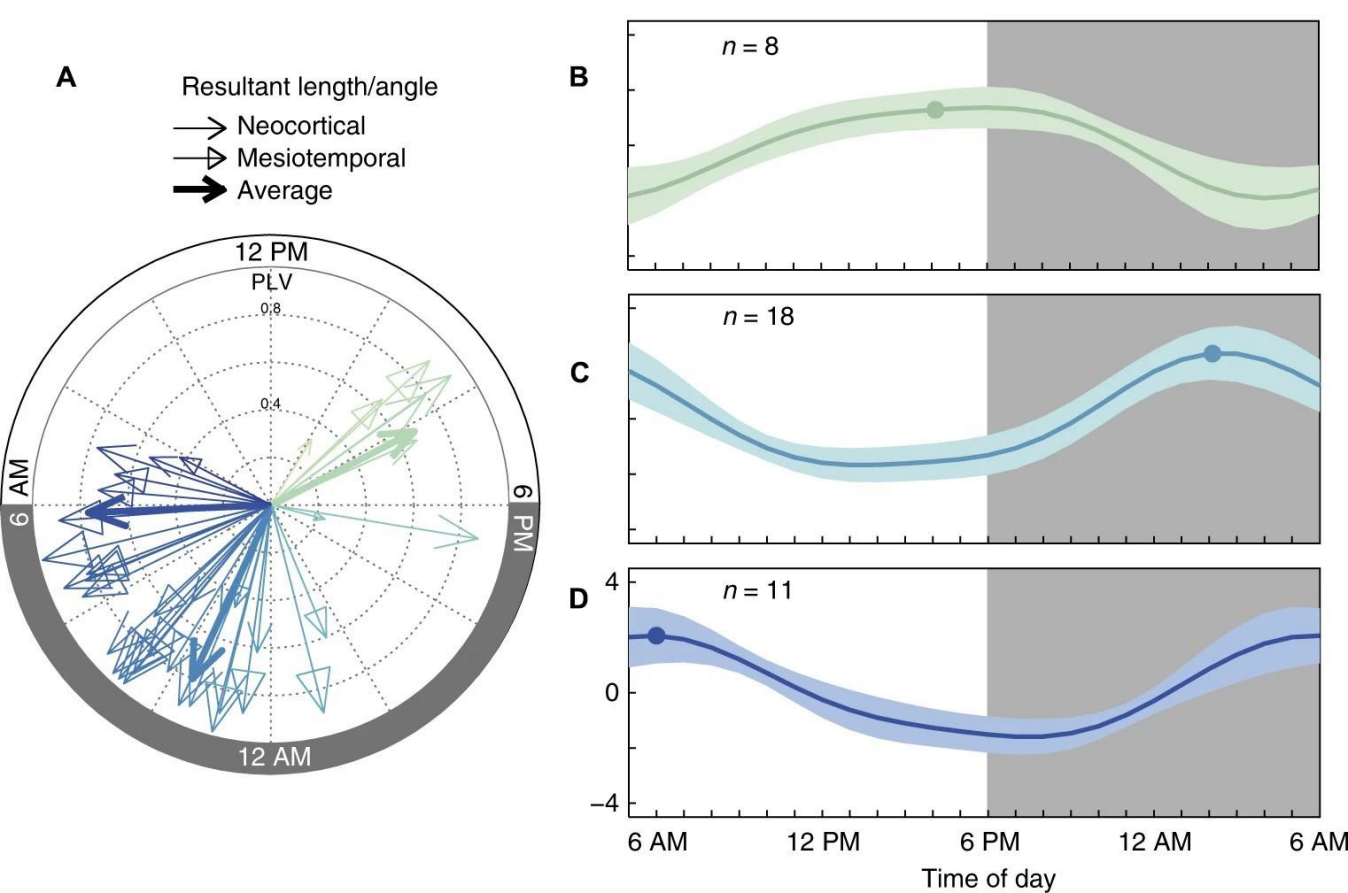
**Circadian rhythm of peak IEA**. (**A**) Each participant’s phase entrainment of the peak IEA circadian rhythm to time of day (N = 37). Based on the time to peak IEA, participants were grouped into three clusters: late afternoon (B), early night (C), and early morning (D). Reproduced under terms of the CC-BY license [13]. Copyright 2018, Baud et al., published by [Springer Nature].

Large clinical trials have demonstrated circadian patterns in epilepsy. Both systemic epilepsy and focal epilepsy have been shown to have a peak onset time in a circadian pattern [15]. The IEA also shows an obvious circadian variation [13] (Fig. 3). Most of the IEA occurs during non-rapid eye movement (NREM) sleep [16]. Circadian changes in the electrophysiology of the neuronal network control the rhythmicity of seizures and IEA. The network activity varies with vigilance states. PWE shows an increased power of a slow-wave activity and sleep spindle during NREM, which contributes to IEA and seizures [17]. The neuronal network activity is significantly regulated by the circadian system, and its molecular mechanism may involve CCGs [18].

**Figure 4.**
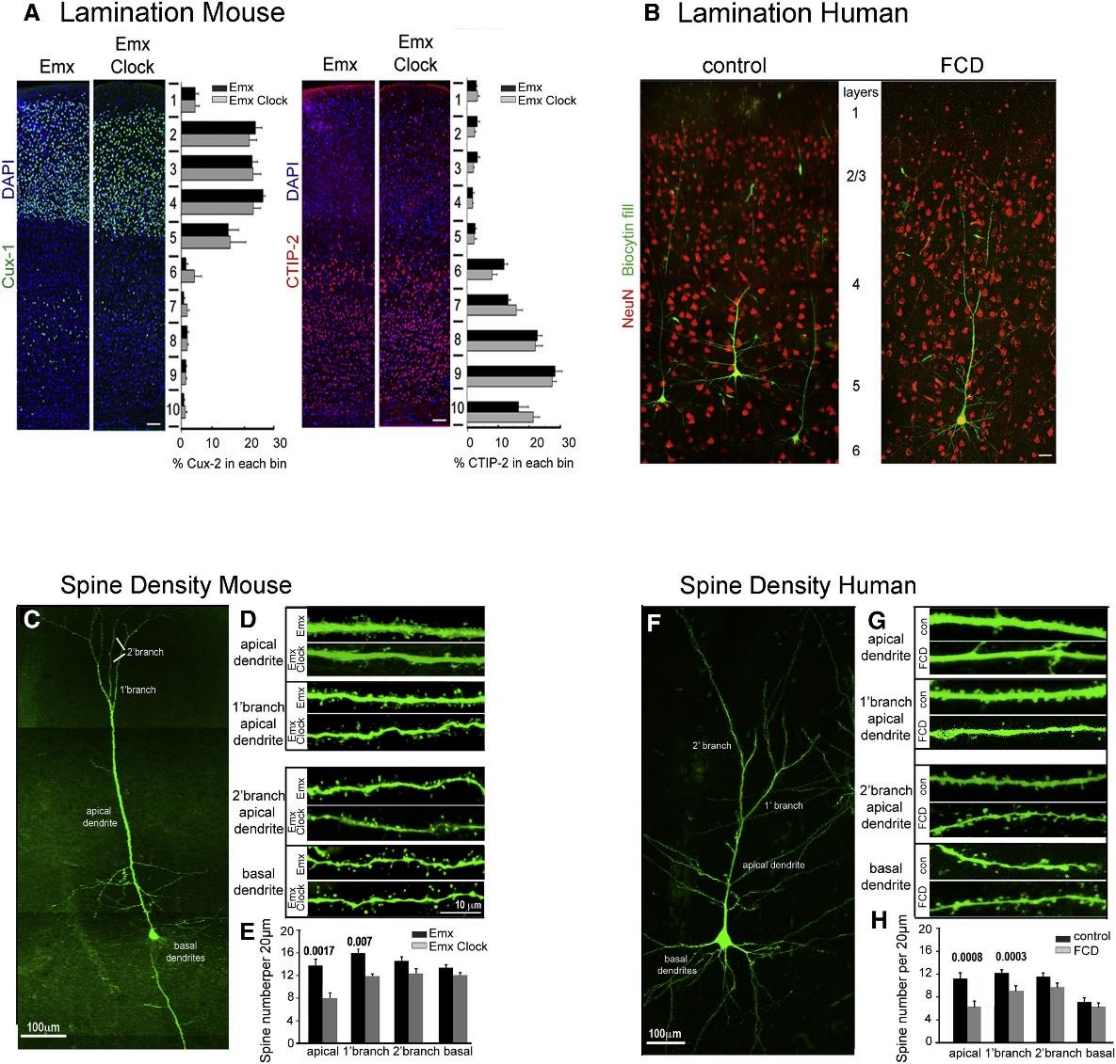
**Neurons from Emx-Cre; CLOCK^flox/flox^ mice, and human epileptogenic tissue**. (**A**) Laminar distribution of somatosensory cortical neurons is normal in Emx-Cre; CLOCK^flox/flox^ mice. (**B**) Epileptogenic tissue from focal cortical dysplasia shows abnormal neuronal density and distribution compared with the control. (**C-E**) Emx-Cre; CLOCK^flox/flox^ mice exhibit specific spine defects in neurons, which is similar to the human epileptogenic tissue (F-H). Reproduced with permission [19]. Copyright 2017, Elsevier.

**Figure 5.**
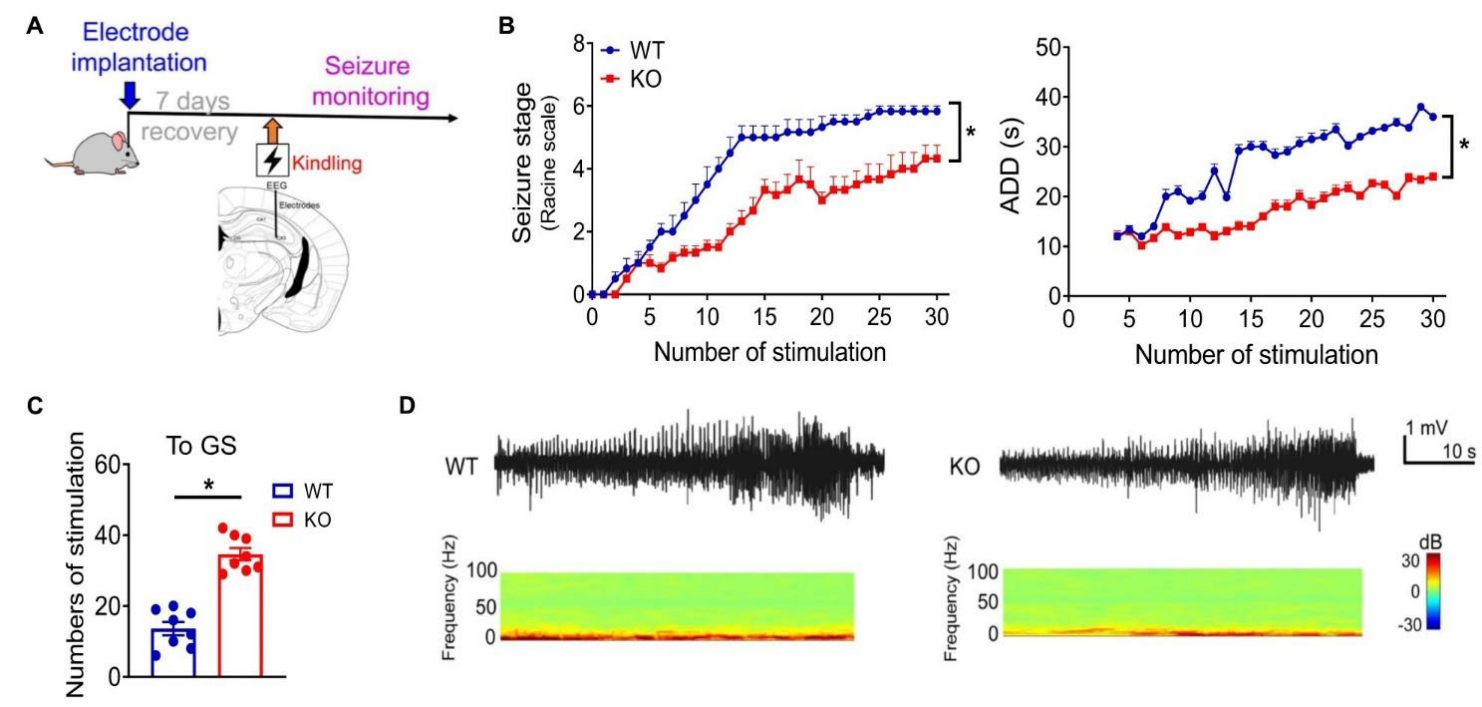
**REV-ERBα knockout alleviates seizures in a hippocampal kindling model**. (**A**) Schematic of the hippocampal kindling model. (**B**) Seizure stages and the after-discharge duration of wild-type and REV-ERBα knockout mice after repeated kindling stimulations. (**C**) Number of stimulations required to achieve generalized seizures in wild-type and REV-ERBα knockout mice. (**D**) EEG recordings and power spectra of wild-type and REV-ERBα knockout mice in a hippocampal kindling model. Reproduced under terms of the CC-BY license.[20] Copyright 2021, Zhang et al., published by [Springer Nature].

Studies on human tissues and animal models have confirmed a dysregulated expression of CCGs in epileptogenic tissues [19, 20]. The dysregulation in turn contributes to epilepsy, which may be related to impaired neuronal networks. By specifically removing the CLOCK gene from the excitatory neurons, it is possible to trigger spontaneous epilepsy and a frequent IEA in mouse models. The neurons lacking CLOCK exhibit abnormalities in the dendritic spines, similar to those observed in human epileptogenic tissues (Fig. 4). Similarly, CLOCK deletion reduced spontaneous inhibitory postsynaptic currents (sIPSCs) and the expression of inhibitory synaptic proteins, thereby increasing neuron excitability [19]. The activation of REV-ERBα increases susceptibility to epilepsy in mouse models. By activating Slc6a1 (Gat1) and Slc6a11 (Gat3), REV-ERBα can enhance the uptake of gamma-aminobutyric acid (GABA) in the synaptic cleft, thereby reducing the inhibitory signal. The ablation of REV-ERBα in mice increases sIPSCs and reduces the vulnerability to seizures [20] (Fig. 5). The downregulation of PAR bZIP transcription factors was observed in rodent models of epilepsy [21]. This downregulation is not dependent on inflammation but rather is mediated by CCGs and hyperexcitability [21]. Mice lacking three PAR bZIP transcription factors (DBP, HLF, and TEF) exhibit susceptibility to spontaneous lethal seizures, potentially attributable to the disruption of negative regulation of homeostatic plasticity [11, 22]. Furthermore, the overexpression of HLF led to a decrease in the frequency and an increase in the amplitude of spontaneous events in neurons under hyperexcitable conditions [21]. Taken together, the normal physiology may be altered in an epileptic brain, leading to changes in the neuronal network activity under a specific time or vigilance state and may create favorable conditions for epilepsy.

#### 3.1.2 Molecular oscillations and rhythmicity of epilepsy (MORE): a more plausible explanation for the circadian rhythm of epilepsy

Although seizures showed a periodic pattern, the peak onset time in the circadian cycle is not always accompanied by seizures. Seizures can also happen beyond the peak period. Therefore, the rhythmicity of a seizure cannot be explained solely by the internal circadian rhythm. The MORE hypothesis may explain the seizure peak hour and address existing questions [23]. As mentioned earlier, the biological clock of our body consists of numerous molecular oscillators and is affected by external factors. Firstly, circadian oscillation can periodically regulate the network activity, causing the neuronal activity in the epileptogenic tissue to periodically get close to the seizure threshold. This provides favorable conditions for epilepsy. For example, a high amplitude-wide slow wave and synchronized oscillations during NREM contribute to IEAs and seizures, whereas asynchronous oscillations during REM prevent seizures [17]. However, this is still not enough to cause seizures. Other internal and external factors, such as insufficient sleep and delayed medication, need to be involved. Therefore, although regarded as an effect of multi-directional factors, the internal rhythm will provide more favorable conditions for seizures in a specific time period, resulting in the peak time for the occurrence of seizures. The circadian rhythm controls cortical excitability. It is also regulated by waking time and increases linearly with the prolongation of waking time [24]. This may explain why sleep deprivation increased susceptibility to epilepsy the next morning. Sleep deprivation increases the cortical excitability based on waking time, and the circadian rhythm promotes the increase in cortical excitability in the morning. The two effects superimposed and induced epilepsy. In addition, external factors may generate a strong signal that is sufficient to change the existing oscillation and lead to seizures, even if the internal rhythm is not suitable.

### 3.2 Clinical potential of targeting circadian rhythm in epilepsy

#### 3.2.1 Accurate prediction of epilepsy - a novel concept based on the circadian rhythm

Seizures are difficult to predict, which often affects the quality of life of PWE and causes a heavy emotional burden. Potential physiological markers, such as stress and hormones, have been used in the algorithm model of seizure prediction [25]. Despite significant advances, the algorithm model cannot be satisfactorily used for seizure prediction. In recent years, studies have reported a promise with the use of circadian and multidien rhythms for seizure prediction. Researchers added the circadian information of seizures into the algorithm model, significantly improving the prediction ability [26]. The circadian and multidien rhythms of EEG signals have also proven to be the most accurate biomarkers for seizure forecasting. Seizures and IEA show similar probability distributions, with seizures consistently happening during the ascending phase of the IEA multidien cycles [27]. Clinical trials have demonstrated that a seizure can be predicted days ahead by utilizing the multidien rhythm of the IEA [28]. As a reliable EEG marker for seizure forecasting, the critical slowdown also caused fluctuations in the circadian and multidien rhythms [29]. It is worth noting that long-term continuous detection through EEG requires invasive procedures, which may not be feasible or acceptable to PWE. Therefore, alternative rhythmic markers, such as heart rate, are being developed, which can be monitored using wearable devices. The heart rate cycle showed a similarity with the multidien rhythm of epilepsy, suggesting its potential in epilepsy prediction [30].

The circadian and multidien rhythms of seizures and the epileptic brain activity show a strong predictive ability of susceptibility to epilepsy, but its implementation and application are still challenging. Regardless of the underlying mechanism, the information about circadian and multidien rhythms in epilepsy will add high value to forecasting.

#### 3.2.2 Chronotherapy benefits PWE

Approximately 30% of PWE have been reported to develop drug-resistant epilepsy. Therefore, there is an urgent need for newer treatments that can effectively reduce seizure frequency and improve seizure- and drug-induced damage to brain function. Based on the interactions between the circadian rhythm and epilepsy, chronotherapy may be a promising treatment option, including the administration of appropriate doses of anti-epileptic drugs (AEDs) at different times and the improvement of the circadian rhythm.

The chronotherapy of epilepsy drugs should integrate pharmacokinetics and seizure times using an AED treatment scheme based on differential dosing times, for example, administering higher doses of AED at periods of higher susceptibility to epilepsy. Clinical trials had initially confirmed the effectiveness of clocking the drugs. Increased dosages of AEDs in the evening enhanced seizure control in individuals experiencing nocturnal and early-morning seizures [31]. The effects of drugs vary with the time of the day due to circadian oscillations in pharmacokinetics (absorption, distribution, metabolism, and excretion) and pharmacodynamics (drug target) [32]. The rhythm of genes related to the metabolism of AEDs also changes in PWE [33]. Therefore, formulating a drug administration plan for a specific time during the day based on chronopharmacokinetics and chrono-pharmacodynamics appears to be a promising approach. We hope that future clinical trials will help support this strategy.

Epilepsy leads to sleep disruptions and circadian misalignments. Further, disturbances in the circadian rhythm and sleep disorders can exacerbate epilepsy, forming a vicious circle [34]. A workshop on epilepsy in 2021 questioned whether seizures can be reduced by improving the circadian rhythm and sleep [35]. We can improve the circadian rhythm of PWE through endogenous signal regulation and external signals. Melatonin is a major regulator of endogenous circadian rhythms. Studies have indicated that epilepsy leads to dysregulation of the expression of melatonin and its receptor [36]. Clinical trials over recent years have demonstrated that melatonin can improve seizures, sleep disorders, and circadian rhythm disorders in PWE [37]. However, it has been observed that various mouse strains do not produce melatonin [38]. Mice lacking melatonin or melatonin receptors demonstrate a normal circadian rhythm [39, 40]. Consequently, the influence of melatonin on the circadian clock may not be as substantial as previously believed. Further investigation is warranted to elucidate the therapeutic mechanism of melatonin in relation to neurological disorders. Improving the circadian rhythm of PWE through external signals, such as diet or light, is also a treatment strategy. The ketogenic diet, characterized by low carbohydrate and high fat intake, has been shown to decrease seizure frequency in mice with epilepsy and restore abnormal circadian rhythms [41]. This effect may be attributed to the activation of peroxisomal proliferator-activated receptor γ (PPAR γ) [42]. PPAR γ can function as a transcription factor, controlling the expression of CCGs [43]. In addition, bright light therapy has been employed as a therapeutic approach for the realignment of endogenous rhythms. Patients undergoing bright light therapy necessitate exposure to intense artificial light for a suitable duration, typically during the morning hours. Bright light therapy was found to reduce the seizure frequency in patients with drug-resistant epilepsy [44]. In a randomized controlled trial, it was also demonstrated that the bright light therapy reduced anxiety and depression in focal epilepsy [45]. However, it is noteworthy that phototherapy may lead to increased seizures in patients with extra temporal focal epilepsy [44]. Additionally, targeting CCGs shows potential as an effective approach for treating seizures. In mouse models, the absence of REV-ERBα or the use of REV-ERBα antagonists (e.g., SR8278) leads to enhanced GABAergic signaling in the hippocampus, reducing vulnerability to both acute and chronic seizures [20]. Nevertheless, further research is necessary to comprehend the use of pharmacological regulators of CCGs in clinical practice.

In conclusion, existing studies have shown that improving sleep and the circadian rhythm can decrease seizures. Despite the lack of adequate evidence clarifying underlying mechanisms and insufficient clinical data, chronotherapy is a promising add-on for AEDs. Nevertheless, additional research is necessary to determine whether chronotherapy is cost effective and efficient with a benefit to patients.

## 4. Ischemic stroke

The circadian rhythm is involved in throughout the processes of ischemic stroke (IS), including its pathogenesis, pathophysiological changes, and manifestation [46].

### 4.1 Circadian variations in IS

Clinical data suggest a circadian variation in IS timing, symptoms, severity, and outcomes. A pronounced circadian rhythm is seen in the onset time of IS, with a significantly higher risk between 6 a.m. and 12 p.m. [3]. A circadian variation was also observed in IS progression. Patients with a nocturnal onset had larger infarct cores and faster infarct growth compared to those with daytime onset [47]. Compared to daytime IS, nocturnal IS is associated with higher severity, earlier neurological deterioration, and a worse functional outcome [48]. The treatment outcome of IS is also related to its diurnal pattern. In patients who experienced acute IS treated with endovascular thrombectomy, those with onsets either between 12 a.m. and 6 a.m. or between 6 a.m. and 12 p.m. appeared to have favorable outcomes 3 months post-treatment [49]. However, these circadian patterns in timing and treatment outcomes of IS may be influenced by multiple factors and cannot accurately reflect the circadian variation in the pathophysiology of IS. For instance, the timing of the symptoms observed may not coincide with the time of the IS onset. Wake-up strokes comprise 14% of IS requiring a visit to the emergency department [50]. Partial wake-up strokes show a shift in the onset time from night to dawn, as it is discovered the next morning and may contribute to IS aggregation. In addition, the effect of circadian rhythms on IS treatment outcomes may be influenced by the “nonworking hours effect”. Lower thrombolysis rates and longer onset-to-treatment times during night hours and nonworking hours than during working hours are noted for patients with IS [51]. This affects the treatment outcomes between daytime and nighttime strokes. Further investigations in epidemiology and new clinical trials are needed to clarify the circadian effect in IS physiology.

Circadian rhythm biomarkers are also disrupted in patients with IS. Studies have reported reduced melatonin levels at night in the serum and urine of patients having an acute IS [52]. Cortisol levels are higher among patients with IS [52]. CCGs including BMAL1, REV-ERBα, PER1, and PER3 exhibited abnormal expression levels in patients with acute IS [53]. Thus, IS can disrupt the circadian rhythm.

**Figure 6.**
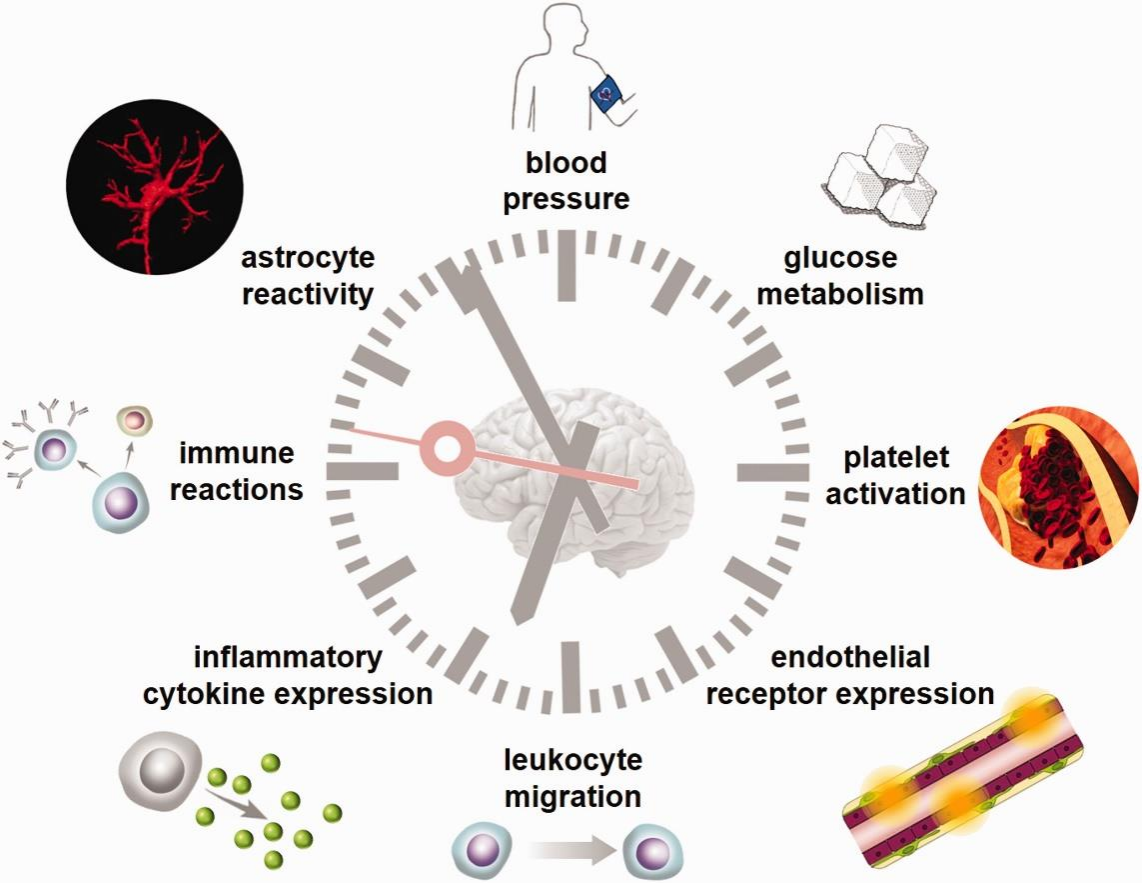
**Circadian rhythms modulate multiple pathophysiological pathways associated with IS**. Circadian rhythms influence the pathophysiology of IS, including blood pressure, glucose metabolism, platelet activation, endothelial receptor expression, leukocyte migration, inflammatory cytokine expression, immune reactions, and astrocyte reactivity. Reproduced under terms of the CC-BY license [175]. Copyright 2021, Boltze et al., published by [SAGE Publications].

### 4.2 Underlying mechanism between the circadian rhythm and IS

The circadian rhythm is involved in multiple pathophysiological pathways associated with IS (Fig. 6). The circadian system regulates cardiovascular risk factors. For instance, sympathetic activity, coagulation activity, and blood pressure are increased in the morning [54]. The cerebrovascular reactivity to carbon dioxide and endothelial function decreased in the morning [55]. These findings indicate that there is a higher risk of IS in the morning. In addition, circadian disruption is associated with outcomes, such as decreased levels of high-density lipoprotein-cholesterol, elevated triglycerides, abnormal release of cortisol, increased blood pressure, and insulin resistance, all of which may potentially contribute to the development of IS [56].

Although the pathophysiological mechanisms associated with IS may be intricate, the primary event in the ischemic tissue is hypoxia and reduced energy supply. PER2 regulates the activity of hypoxia-responsive factor 1, a crucial regulator of the hypoxia response [57]. This indicated a possibility of a circadian variation in the brain’s response to ischemia. In the primary neurons of mouse models, the release of glutamate and reactive oxygen species induced by oxygen and glucose deprivation (active phase) was lowered during the night compared to the daytime (inactive phase) [58]. GluA1 expression is lower, and the infarct volume is smaller in mice having a nocturnal IS compared with daytime IS models. The degradation of GluA1 was found to be mediated by PER1 [59]. These data suggested that the pathological activities of IS, such as hypoxia, excitotoxicity, and oxidative stress, are controlled by fluctuations in the circadian rhythm. This may explain the circadian differences in the severity of IS.

IS is accompanied by immune reactions. In the ischemic area of the brain, inflammatory cytokines and chemokines induce the activation of glial cells and recruitment of peripheral leucocytes. Results of a mice model suggested that the circadian rhythm significantly affects the immune responses after focal cerebral ischemia [60]. BMAL1 positively regulates the expression of IL-6 in the microglia under particular pathological conditions. In mouse models with middle cerebral artery occlusion, conditional BMAL1 ablation in CD11b-expressing cells, including microglia, resulted in a decrease in the upregulation of IL-6 expression and a reduction in neuronal damage [61]. BMAL1 deletion decreased the infarct core size and glial activation in a photothrombotic mice model [62]. In addition, BMAL1 may regulate the phenotype, numbers, superoxide-producing capacity, and extracellular traps-forming capacity of the circulating neutrophils that follow strong circadian rhythms [63]. The functional circadian oscillation of neutrophils may contribute to differences in immune responses following IS. Taken together, existing evidence suggests that the circadian rhythmicity of the immune system mediates the pathophysiology following IS.

Angiogenesis is critical for IS recovery. Substantial evidence supports that CCGs are implicated in regulating the process [64]. For example, BMAL1 promotes vascular endothelial growth factor (VEGF) expression, leading to the circadian rhythmicity of angiogenesis [65]. PER2 and CRY1 periodically inhibit hypoxia-induced vascular endothelial growth factor transcription [66]. This may enable choosing the most appropriate timing for the administration of angiogenic agents.

### 4.3 Optimizing the timing of therapies via the circadian variations of IS

Chronotherapy becomes especially important when the disease's risk and severity fluctuate throughout time. The circadian variations in the risk factors of IS hold a promising strategy to determine the optimal timing for administering drugs (such as antihypertensive drugs and anticoagulation), thereby improving the therapeutic effects [67]. For example, the administration of routine blood pressure-lowering medications at bedtime instead of waking, improved ambulatory blood pressure and lowered the risk of IS [68]. Platelet antagonists, such as aspirin, have been recommended for the evening [69]. This may be beneficial for achieving optimal platelet inactivation the next morning (the time of highest thromboembolic risk). However, the therapeutic effects of nighttime aspirin administration have yet to be explored in clinical trials. Further research is needed to determine the potential benefits of adjusting drug doses based on circadian variations for coagulation function and IS risk factors. Circadian variations in the progression of IS suggest that the therapeutic windows may be shorter at certain times [46]. Therefore, some treatments need to be administered sooner within the onset. Given that the circadian rhythm is involved in regulating various pathophysiological mechanisms of IS, some components of the circadian system may be therapeutic targets for IS. Studies have confirmed that exogenous melatonin administration attenuated ischemic stroke injury, improved neurological deficits, and protected the integrity of the blood-brain barrier (BBB) [70, 71]. REV-ERBα agonists (e.g., SR9009), acting as pharmacological regulators of the circadian rhythm, inhibited neuronal apoptosis during early brain injury after subarachnoid hemorrhage [72].

As suggested previously, the development of circadian-driven precise treatments for IS nucleates novel ideas for the timing of drug administration, thereby contributing to promising clinical outcomes eventually. Further research is necessary to enhance our comprehension of the function of circadian rhythm in the pathophysiology of IS, aiming to improve the treatment and care of patients.

## 5. Alzheimer’s disease

The available evidence reveals a reciprocal connection between the circadian rhythm and Alzheimer’s disease (AD), suggesting that circadian dysfunction might have a significant impact on the advancement of AD. Thus, the circadian system has become a compelling focus for AD research.

### 5.1 Circadian disruption in AD

Multiple functional indicators of the circadian rhythm are disrupted in patients with AD. Patients with preclinical AD had fragmented rest-activity rhythms, which means the physical activity is spread across 24 hours [73]. Patients with moderate-to-severe AD showed more pronounced disruptions, including higher levels of fragmentation in the rest-activity rhythm and a reduced rhythm amplitude [74]. Patients with AD also lack a clear sleep-wake rhythm, which is primarily manifested as increased nocturnal wakefulness, irregular daytime sleep, and a phase delay in bed and wake times [75]. One study reported that the circadian rhythm of the body temperature in AD patients completely disappeared [76]. In addition, Patients with AD also exhibited dysregulated rhythms of melatonin and cortisol [77]. The disrupted temperature and hormone rhythms may contribute to the sundowning phenomenon, which justifies the worsening of neuropsychiatric symptoms in patients with AD around sunset [78].

It has been confirmed that the core components of the circadian system have changed in patients with AD. AD patients showed loss of ipRGCs and optic-nerve degeneration [79]. Additionally, patients with AD show high degradation of the SCN, including accumulation of tau neurofibrillary tangles and loss of neurons expressing vasopressin, vasoactive intestinal peptide, and neurotensin [80]. This would result in disrupted SCN timing and reduced SCN circadian outputs. Significant changes in the pattern of CCG rhythms were also noted in the AD brain [81]. The rhythmic methylation of BMAL1 is impaired in AD [82]. Many studies have shown that BMAL1 dysfunction and AD pathology are mutually promotional. Amyloid-β (Aβ) induced the degradation of BMAL1 in a mouse model [83]. Global BMAL1 ablation increased the accumulation of amyloid plaques [84]. The mice model of tauopathy also exhibited disruption of circadian clock function [85]. However, recent studies indicate that the specific deletion of BMAL1 in astrocytes had no impact on plaque accumulation in a mouse model of AD [86]. Moreover, astrocyte-specific BMAL1 deletion in mouse models of tauopathy or α-synucleinopathy reduced both tau and α-synuclein aggregation as well as the associated pathology [87]. The results of previous research do not align with these findings, suggesting that the pathological exacerbation of AD resulting from global BMAL1 deletion is independent of astrocytes. The intricate relationship between AD and CCGs remains multifaceted and not fully comprehended. Although the data demonstrate a strong correlation between AD and circadian disorders, further research is necessary to comprehend the fundamental mechanism.

### 5.2 Underlying mechanism of circadian disruption that exacerbates AD

Preliminary evidence suggests that circadian disruption may be a potential risk factor for developing AD. For instance, BMAL1 dysfunction blunts Aβ rhythms in the interstitial fluid and accelerates Aβ accumulation [84]. Here, several promising hypotheses are briefly discussed.

Circadian disruption may aggravate the accumulation of pathological protein. At first, scholars proposed that circadian disruption leads to imbalanced proteostasis (protein homeostasis) and the accumulation of misfolded proteins [88]. Proteostasis encompasses biogenesis, folding, trafficking, and degradation of proteins. The circadian rhythm may regulate proteostasis. The analysis of gene expression in the SCN showed that genes related to protein stabilization and folding are rhythmically expressed, such as heat shock proteins HSP70 and HSP90 [89]. Although not well elucidated, proteostasis disruption is expected to enhance our comprehension of the connection between circadian dysfunction and AD. Secondly, neuroinflammation contributes to Aβ deposition and drives the pathogenesis of AD [90]. Circadian dysfunction can drive neuroinflammation. BMAL1 deletion induces the expression of genes associated with inflammation [91]. The ablation of REV-ERBα increased spontaneous microglial activation, nuclear factor-kappa B signaling activation, and secondary astrogliosis [92]. Thus, circadian dysfunction may aggravate the pathological progression of AD by driving neuroinflammation.

In addition, circadian disruption may impair the clearance of pathological protein. Recent studies have shown that the glymphatic system clears metabolites and soluble proteins (like Aβ and tau) from the central nervous system through perivascular spaces [93]. Glymphatic function is regulated by the circadian rhythm, which is active during sleep and inactive during wakefulness [94]. Furthermore, the activity of the glymphatic system positively related to the proportion of slow-wave sleep (SWS) in total sleep [95]. SWS deprivation (SD) increases Aβ deposition and tau pathology [96]. However, partial SD with preserved SWS had no effect on Aβ deposition [97]. It suggests that SWS is critical to Aβ clearance. SWS impairment is commonly seen in patients with AD [98]. Thus, available evidence suggests that circadian dysregulation can lead to glymphatic dysfunction, thus impairing the removal of Aβ.

The BBB is a specialized tissue structure in the central nervous system that plays a vital role in maintaining brain homeostasis. BBB disruption has been reported to be associated with the development and progression of AD [99]. Impaired BBB results in reduced Aβ clearance, increased circulating Aβ influx, and processing of the Aβ precursor protein [100]. Furthermore, dysfunction of the BBB has been linked to increased production of Aβ [101]. Recent findings indicate that the biological clock controls the function of the BBB [102]. Aβ clearance across the BBB follows a circadian rhythm, with increased clearance occurring during sleep [103]. Circadian disruption may impair the function of the BBB. For example, BMAL1 disruption impairs BBB integrity via pericyte dysfunction [104]. Therefore, circadian dysregulation may contribute to the impairment of the BBB, ultimately worsening the AD pathology.

### 5.3 Chronotherapy improves cognitive function in patients with AD

Given that circadian dysregulation plays an important role in the pathophysiology of AD, the development of chronotherapy in AD treatments should be given increased focus. Here, we discuss several encouraging chronotherapies that potentially function by promoting clock synchronization and enhancing the amplitude of the rhythm.

Melatonin and light therapy are the two most relevant chronotherapies for AD. Numerous clinical trials have demonstrated the benefits of melatonin individuals with AD [105]. The French Medical and Research Sleep Society concluded that melatonin could help patients with AD by improving sleep quality and regulating sleep-wake cycles [106]. To date, several clinical trials have identified that bright light therapy can improve AD symptoms, particularly when given in the morning. Bright light therapy can stabilize circadian rhythms, promote sleep, and improve cognition [107]. These beneficial effects may be due to the strong entraining effects of light on the SCN. Gamma entrainment using sensory stimuli (GENUS) is another perspective that requires investigation. Cognitive function is believed to be associated with the brain's gamma-band activity, which ranges from 30 to 80 Hz. Of note, deficits in gamma band oscillations have been observed in AD brains [108]. Recently, GENUS has been considered for AD treatment; 40Hz combined visual and auditory stimulations have been proven to improve daily activity, rhythmicity, and cognition in patients [109]. However, clinical trials have also reported a lack of benefit with melatonin and light therapy [110]. Despite contrasting data, research supports the therapeutic value of melatonin and light therapy [111, 112]. Other external zeitgebers (such as diet and exercise) can also act on the circadian system. For instance, researchers used a beverage containing omega-3 fatty acids as an additional treatment for AD patients receiving cholinesterase inhibitors. The omega­3 fatty acid may delay dementia and upregulate the CLOCK and BMAL2 (a paralog of BMAL1) [113, 114]. Aerobic exercise can help downregulate cortisol levels to reduce the sundowning syndrome in AD [115].

To date, a variety of agonists, antagonists, or stabilizers targeting the major components of the circadian system and their regulatory molecules have been identified. Targeting the circadian rhythm has been a priority of the investigation of newer AD therapies. The inhibition of REV­ERBs enhanced BMAL1 transcription and Aβ clearance in microglial cells [116]. CK1δ and CK1ε are key kinases for the phosphorylation (inactivation) of the circadian protein PER [117]. Importantly, CK1δ and CK1ε are highly overexpressed in the brains of patients with AD [118]. The small molecule CK1δ/ε inhibitor (PF-670462) reversed pathological alterations and rescued circadian disturbances in an AD mouse model [119]. Glycogen synthase kinase 3 (GSK3) phosphorylates CCGs and shows a circadian variation [120]. The chronic activation of GSK3 has been detected in AD, which aggravated the pathophysiology and disrupted the rhythms of CCGs [121]. The inhibitor of GSK3β (Chir99021) prevented the glial specification induced by Aβ [122]. Additionally, small molecule therapies have many advantages, such as rapid/immediate effects on protein functions, reversible and conditional controls in any biological systems, highly penetrant effects, the ability to easily combine with other treatments, and a specific impact on a relevant mechanism [119]. In conclusion, studies that detect the efficacy of a pharmacological modulator to correct circadian disruption in AD are worthy of consideration.

Generally, a growing body of evidence supports that chronotherapy improves sleep quality and cognitive function of patients with AD, implying the add-on benefit of chronotherapy. However, previous research has primarily concentrated on the stages subsequent to the initial manifestation of a condition. Testing the role of chronotherapy for AD prevention at the preclinical stages may be more beneficial.

## 6. Parkinson’s disease

Circadian fluctuations have been noticed in several Parkinson’s disease (PD) symptoms [123]. Although numerous studies have demonstrated circadian dysfunction in patients with PD, there is a need for further discussion and investigation into the correlation between PD and the circadian system.

### 6.1 Circadian disruption in PD

**Figure 7.**
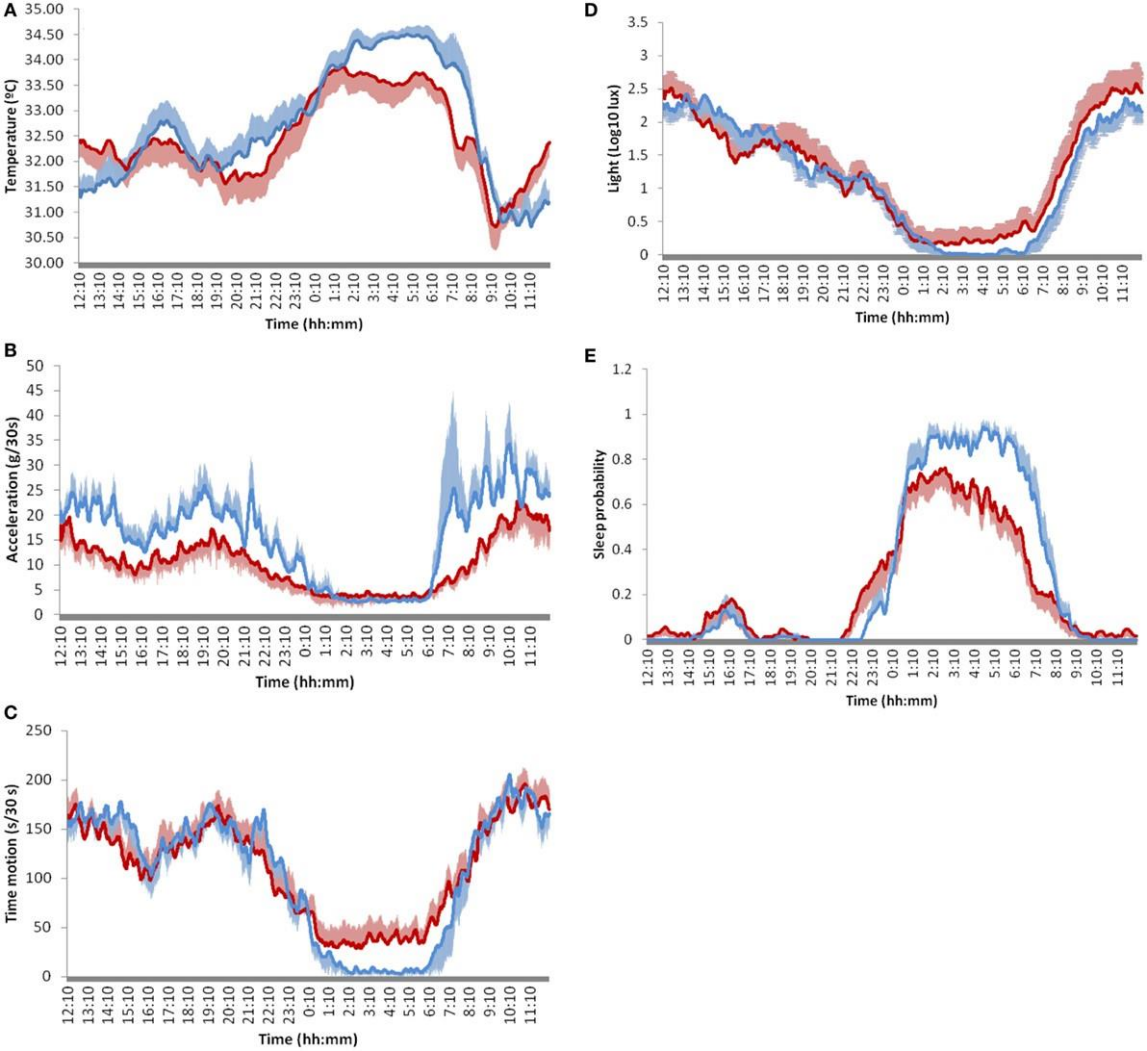
**Comparison of 24-hour mean waveforms of circadian variables in patients with PD (red) and controls (blue)**. (**A**) skin temperature, (B) acceleration, (C) movement duration, (D) light exposure, and (E) sleep probability. Reproduced under terms of the CC-BY license [125]. Copyright 2018, Navarro et al., published by [Frontiers].

Sleep-wake disturbances, including insomnia, daytime sleepiness, and REM sleep behavior disorders, are prevalent non-motor symptoms among individuals diagnosed with PD [124]. Researchers used actigraphy to detect the physical activity in patients with PD over several days. Patients showed a reduction in the amplitude of circadian rhythms compared with controls [125] (Fig. 7). A phase shift also occurred with the progression of PD [126]. The association of circadian disruption with the autonomic nervous system is also recognized in patients with PD, mainly manifesting as the circadian disruption of blood pressure and decreased heart rate variability [127]. Furthermore, patients with PD exhibited significantly elevated levels of plasma cortisol concentrations. Patients with PD exhibited a reduction in the circadian fluctuation of cortisol levels [128]. Patients with PD had lower serum melatonin levels compared with controls [129]. However, contradictory findings were reported in other studies [130]. The variation in the study population and methods could potentially account for this.

Anatomically, reduced density and dendritic complexity of ipRGCs have been observed in patients with PD [131]. These pathological changes could potentially lead to the deterioration of the circadian output. In addition to the impairment of ipRGCs and SCN, the widespread neuropathological changes in PD may disrupt the signal networks between SCN and other brain regions, leading to circadian dysregulation [132]. At the molecular level, the peripheral expression of BMAL1, CLOCK, CRY1, PER1, and PER2 was decreased in patients with PD [129]. In a PD mouse model, the expression of REV-ERBα was reduced and was accompanied by the disappearance of circadian oscillations [133]. Although the related research is not thorough, the existing evidence showed that the impairment of SCN and the expression of CCGs is the basis of the circadian disruption in PD.

### 6.2 Role of dopamine in circadian disruption in PD

Dopamine serves as a regulator of the circadian system. Evidence showed that the circadian release of dopamine contributes to light adaptation and transmission of light information to the SCN through ipRGCs [134]. Dopaminergic retinal cell loss has been observed in patients with PD, which may have implications on external light cues toward circadian disruption [135]. In a mouse model of PD, dopamine loss was shown to disrupt the circadian expression of CCGs, suggesting a pathophysiological involvement in PD [136, 137]. In a mouse model, dopaminergic impairment in the substantia nigra pars compacta decreased dopamine and changed the circadian rhythm of melatonin in the striatum [138]. Therefore, an impaired dopaminergic signal may implicate changes in the expression rhythms of CCGs, leading to circadian disruption among patients with PD.

Given the relationship between dopamine and the circadian rhythm, dopaminergic medications may have an effect on the circadian system in PD. Some investigations reported that dopaminergic medication increases melatonin secretion in patients with PD [139]. However, the patients receiving dopaminergic treatment showed a phase difference between the melatonin rhythm and sleep time, indicating a disconnection between the circadian rhythm and sleep control regulation [140]. Furthermore, animal experiments showed that long-term levodopa therapy may further aggravate circadian disruption in PD [141]. This is an interesting phenomenon. In view of these results, it is necessary to further study the effect of dopamine therapy on the circadian rhythm in PD. This will advance our knowledge about the relationship between circadian function, dopamine, and PD.

### 6.3 Chronotherapeutic interventions in PD

Chronotherapy to improve circadian function in PD represents a novel strategy. Chronotherapeutic interventions have been demonstrated to have beneficial impacts on the sleep, mood, and motor abilities of patients with PD [142].

Given that the degeneration of ipRGCs in patients with PD can alter susceptibility to external light signals, light therapy is a promising strategy for managing circadian disruption in PD. Several studies have reported the effectiveness of light therapy on sleep impairment [143]. Participants in these trials were administered bright light treatment ranging from 1,000 to 10,000 lux. The treatment was administered upon waking up and/or prior to going to sleep. Results of these clinical trials have demonstrated that light therapy improved daytime sleepiness, insomnia, and nighttime awakenings in patients with PD. Bright light therapy also appears to be beneficial for depressive and motor symptoms in PD [143, 144]. Further research is required to determine the most suitable timing, duration, frequency, and parameters of light therapy. Furthermore, preliminary clinical trials elucidated the benefits of melatonin in PD. Melatonin increased the levels of BMAL1 in patients with PD to restore the circadian rhythm [145]. Melatonin may improve sleep health, non-motor symptoms, and the quality of life of patients [146]. In addition to restoring the circadian rhythm, melatonin also has a variety of effects, such as antioxidant and anti-inflammatory, beneficial to patients with PD [147]. Although there are clinical trials showing contrasting results, the chronotherapeutic effects of melatonin are still worth investigating in patients with PD [148]. As the external zeitgeber, exercise training has been also proven to improve motor symptoms, sleep disorders, and cognitive function in patients with PD [149].

Treatment with light therapy and exogenous melatonin appears to have positive effects on sleep quality and motor function in patients with PD [142]. These findings suggest a promising role for chronotherapy in PD. Due to the heterogeneity of the outcomes and a small number of participants, further large and well-designed trials are required to clarify the merits of circadian-based interventions in patients with PD.

## 7. Primary headache

According to clinical reports, some patients experience diurnal variations in the onset of headache. In this section, we discuss circadian relevance in cluster headache (CH) and migraine. We further describe the existing pain medicines, potential therapeutic options, and novel clock-modulating compounds.

### 7.1 Circadian relevance in CH and migraine

Data from clinical trials have shown a distinct circadian pattern in CH. Most patients with CH have symptoms at the same time every day, which appear to peak at night [150]. One critical nexus between the circadian system and CH is the SCN. Neuroimaging showed the activation of the hypothalamus, including SCN, during CH attacks [151]. According to recent research, there is a significant connection between CH and single nucleotide polymorphisms in CCGs, with the involvement of CLOCK variant rs12649507 and the CRY1 variant rs8192440 [152]. Decreased REV-ERBα expression has also been found in patients with CH [153]. On a circadian scale, the pain, intensity, and frequency of migraine peak in the morning [150]. Impaired rest-activity rhythms were observed in migraine patients. Patients with migraine showed decreased daily activity and more fragmented sleep over a 24-hour period [154]. In addition, the rhythmicity of melatonin and cortisol is impaired in patients with migraine [155, 156]. Like CH, patients having migraine show an early activation of the hypothalamus and SCN. CK1δ mutations were seen in familial migraine [157]. An association was observed between the RORα variant rs4774388 and migraine [158]. Neuropeptides, such as vasoactive intestinal peptide, pituitary adenylate cyclase-activating peptide, and orexin, are modulated by the SCN and associated with CH and migraine. Thus, CH and migraine exhibit connections to the circadian system across various aspects, encompassing behavior, neuroanatomy, molecular biomarkers, and transcriptomics.

**Figure 8.**
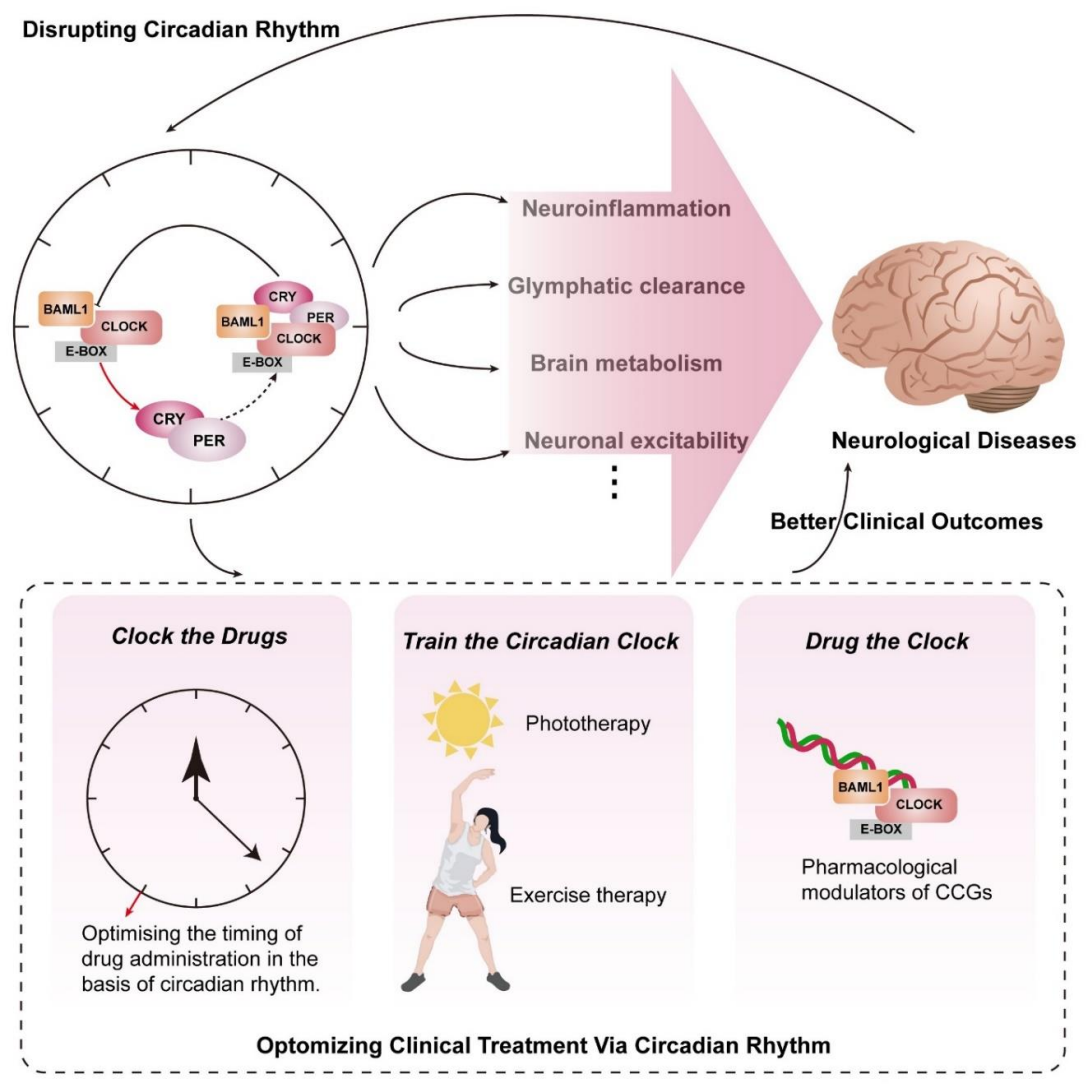
**Circadian-based interventions improve the clinical outcome of neurological diseases**. There is a bidirectional relationship between circadian rhythms and neurological diseases. Circadian rhythm could regulate the severity and progression of neurological diseases through a variety of pathways. Neurological diseases can lead to the disruption of circadian homeostasis. Circadian-based interventions, including clocking the drug, training the circadian clock, and drugging the clock, can offer better clinical outcomes for patients with neurological disease.

**Table 1:** Underlying mechanism between the circadian rhythm and neurological diseases.

Diseases	Underlying mechanism between the circadian rhythm and neurological diseases	References
**Epilepsy**	1. The circadian changes of the neuronal network control the rhythmicity of seizures and IEA.	[17, 165]
	2. The CCGs disruption decreases the inhibitory signal, contributing to seizures.	[11, 19, 20, 166]
**IS**	1. The circadian system regulates IS risk factors, leading to a higher risk of IS in the morning.	[54, 55, 167, 168]
	2. Circadian disruption increases cardiovascular risk factors, promoting IS occurrence.	[56, 169, 170]
	3. CCGs regulate the response to hypoxia, inflammatory responses and angiogenesis.	[57, 59, 61-63, 65, 66, 171-173]
**AD**	1. The loss of ipRGCs and SCN degeneration disrupted sleep-wake cycles and rest-activity rhythms in patients with AD.	[79]
	2. The dysregulation of CCGs increased the accumulation of Aβ plaques.	[84]
	3. Circadian disruption leads to imbalanced proteostasis and the accumulation of misfolded proteins.	[88]
	4. Circadian dysfunction drives neuroinflammation to aggravate AD pathology.	[90-92]
	5. Circadian disruption may impair the clearance of the pathological protein.	[93, 94]
**PD**	1. The dysregulation of ipRGCs decrease the circadian output in patients with PD.	[125]
	2. Dopaminergic damage disrupts the expression rhythms of CCGs, leading to circadian disruption in PD patients.	[137, 138, 174]

AD, Alzheimer’s disease; Aβ, Amyloid-β; CCGs, clock-controlled genes; IEA, interictal epileptiform activity; ipRGCs, intrinsically photosensitive retinal ganglion cells; IS, ischemic stroke; SCN, suprachiasmatic nucleus; PD, Parkinson’s disease.

### 7.2 Chronotherapy improved the efficacy in the treatment of CH and migraine

Zeitgebers, such as behavior, diet, and environment, can affect the circadian rhythm. Bright light therapy and the appropriate timing of drug administrations are employed in clinical trials to treat migraine [159]. For example, the administration of onabotulinum toxin-A in the afternoon appears beneficial for increasing the effectiveness of the preventive treatment of migraine [159]. In addition, at least five drugs, including melatonin, corticosteroid, lithium, verapamil, and valproic acid, are approved for the treatment of CH and can act on the circadian system. As biomarkers, melatonin and corticosteroid can reset the circadian rhythms. Lithium and verapamil affect the expression of genes modulating the circadian clock [160]. Valproic acid may inhibit histone deacetylation to affect the timing of PER2 expression [161].

To summarize, the circadian rhythm seems to have an impact on headaches and this association can be applied to develop therapeutic strategies for headaches. Circadian-based interventions may be beneficial to alleviate or manage primary headaches. However, our understanding of chronotherapy in primary headaches is restricted due to a limited number of participants and the absence of preclinical trials. We believe that research using longitudinal cohorts can bring new insight into this unexplored area.

## 8. Conclusion and future perspectives

Advances in the research on the circadian rhythm present tremendous opportunities as well as challenges in neurological diseases. Deciphering the precise relationship between the circadian system and neurological diseases contributes to comprehending the clinical manifestations and the development of novel therapeutic approaches. Numerous clinical and experimental studies have revealed that the circadian system modulates many aspects of the pathophysiology of neurological disorders, such as cortical excitability, immune response, and pathological protein synthesis. The underlying mechanisms between the circadian rhythm and neurological diseases are summarized in Table 1. In addition, patients with neurological diseases show rhythm disorders of melatonin, cortisol, and CCGs. The dysfunction in turn disrupts the circadian rhythm, with changes, such as reduced circadian amplitude, and increases the risk of occurrence and progression of neurological diseases. From these results, we can say that interventions targeting the circadian rhythm have the potential to affect neurological diseases. Differential timings of medication doses, circadian rhythm improvement by zeitgeber, and pharmaceuticals targeting CCGs are expected to improve outcomes and perhaps even slow down the progression of these disabling neurological diseases (Fig. 8). The circadian-based interventions for clinical treatment of neurological diseases are summarized in Table 2. More efforts are required to develop novel chronotherapy options in the future, which will bring hope to more patients. Although numerous studies provide promising data for clinical translation, several challenges remain. First, we need to identify a “perfect” circadian biomarker. It may be detected using a wearable device or a blood test [162]. Second, clinical trials on the circadian rhythm necessitate specific protocols (such as consistent light-dark cycles, bed/wake times, and feeding/fasting times), which may be challenging for patients to adhere. Third, at a clinical level, how circadian information assists patient management remains to be elucidated further. Personalized treatment for each patient is necessary considering the differences in chronotypes (individual circadian timing) of each patient.

**Table 2:** Circadian-based interventions can optimize the clinical treatment of neurological diseases.

Diseases	Optimizing clinical treatment	References
**Epilepsy**	1. Circadian information improves the prediction ability. 2. The rhythm of EEG signals is a promising biomarker for seizures. 3. Clock the drugs: integrating pharmacokinetics and seizure times based on differential dosing times. 4. Formulating drug administration plans based on chronopharmacokinetics and chronopharmacodynamics. 5. Regulating endogenous/external signals of the circadian rhythm (eg., melatonin, diet and light) can reduce the seizures. 6. Targeting CCGs (eg., REV-ERBα) is a promising therapeutic target in epilepsy.	[20, 26, 27, 31-33, 37, 44, 45]
**IS**	1. Choose the most appropriate time for drug administration based on circadian variations in the risk factors of IS. 2. Shorter therapeutic windows induced by circadian variations. 3. Training the circadian clock (eg., melatonin) or targeting CCGs (eg., REV-ERBα) can improve the prognosis of patients with IS.	[46-49, 67, 72]
**AD**	1. Training the circadian clock (eg., melatonin, light therapy, diet and exercise) can improve AD symptoms. 2. GENUS has been shown to improve daily activity, rhythmicity, and cognition in patients with AD. 3. Targeting CCGs (eg., REV­ERBs, CK1δ/CK1ε, GSK3)	[106, 107, 109, 115-117, 121]
**PD**	1. Light therapy can improve sleep quality and depressive/motor symptoms. 2. Melatonin can improve sleep health and non-motor symptoms. 3. Exercise can improve motor symptoms, sleep disorders, and cognitive function.	[143, 146, 149]
**Primary headache**	1. Clock the drugs: appropriate timing of drug administrations (eg., onabotulinum toxin-A in afternoon) can increase the pharmacotherapy. 2. Drugs acting on the circadian system (e.g., melatonin, corticosteroid, lithium, verapamil, and valproic acid) are effective treating headache. 3. Bright light therapy can alleviate headaches.	[159-161]

AD, Alzheimer’s disease; CCGs, clock-controlled genes; EEG, electroencephalogram; GENUS, gamma entrainment using sensory stimuli; GSK3, Glycogen synthase kinase 3; IS, ischemic stroke; PD, Parkinson’s disease.

In addition, many circadian rhythm studies have been conducted in nocturnal rodents, specifically rats and mice. However, it should be noted that laboratory rodents are mainly nocturnal animals, while humans are diurnal [163, 164]. Experiments conducted during the daytime of human activity are consequently performed during the inactive phase of rodents and the active phase of humans. This difference may contribute to the translational failure. For example, some neuroprotective approaches improved pathological changes only in daytime (inactive phase) rodent models of IS [58]. But most IS in clinical trials occur during the daytime (active phase in humans). It may lead to the ineffectiveness of neuroprotective strategies proven efficacious in rodent IS models when applied in clinical trials. Therefore, the reverse oscillation of circadian rhythm in humans and nocturnal rodents must be considered when conducting translational studies on neurological diseases.

In conclusion, the circadian rhythm is involved in the complete pathophysiological process of central neurological diseases. Therefore, acknowledging the significance of circadian rhythms in neurological disorders and applying the findings and principles to develop therapy and management strategies will have profound and transformative implications. Further research is needed to ensure that circadian-based interventions are applicable to a wider group of patients with neurological diseases.
